# Systemic Deficiency of GHR in Pigs leads to Hepatic Steatosis via Negative Regulation of AHR Signaling

**DOI:** 10.7150/ijbs.64894

**Published:** 2021-10-03

**Authors:** Qi Han, Huiling Chen, Likai Wang, Yang An, Xiaoxiang Hu, Yaofeng Zhao, Hao Zhang, Ran Zhang

**Affiliations:** 1 State Key Laboratory of Agrobiotechnology, College of Biological Sciences, China Agricultural University, Beijing, 100193, China.; 2 MD Department of Plastic Surgery, Peking University Third Hospital, Beijing, 100191, China.; 3 National Engineering Laboratory for Animal Breeding, China Agricultural University, Beijing, 100193, China.

**Keywords:** Laron syndrome, GHR, hepatic steatosis, AHR

## Abstract

Laron syndrome (LS) is an autosomal recessive genetic disease mainly caused by mutations in the human growth hormone receptor (*GHR*) gene. Previous studies have focused on *Ghr* mutant mice, but compared with LS patients, *Ghr* knockout (KO) mice exhibit differential lipid metabolism. To elucidate the relationship between *GHR* mutation and lipid metabolism, the role of GHR in lipid metabolism was examined in *GHR* KO pigs and hepatocytes transfected with si*GHR*. We observed high levels of free fatty acids and hepatic steatosis in *GHR* KO pigs, which recapitulates the abnormal lipid metabolism in LS patients. RNAseq analysis revealed that genes related to the fatty acid oxidation pathway were significantly altered in *GHR* KO pigs. AHR, a transcription factor related to lipid metabolism, was significantly downregulated in GHR KO pigs and si*GHR-*treated human hepatocytes. We found that AHR directly regulated fatty acid oxidation by directly binding to the promoters of ACOX1 and CPT1A and activating their expression. These data indicate that loss of GHR disturbs the ERK-AHR-ACOX1/CPT1A pathway and consequently leads to hepatic steatosis. Our results established AHR as a modulator of hepatic steatosis, thereby providing a therapeutic target for lipid metabolism disorder.

## Introduction

Laron syndrome (LS), also known as growth hormone insensitivity syndrome, is an autosomal recessive genetic disease associated with severe postpartum growth retardation 1. Single gene deletion of *GHR* or a lack of the molecule downstream of the receptor in the GH signalling pathway are the causes of LS. Among these causes, *GHR* mutation is the most common genetic aberration 2,3. GH binds to dimeric GHR to induce its conformational rotation, which leads to the activation of different signalling pathways, including the JAK2-STATs 4, MAPK 5 and PI3K-AKT 6 pathways, to promote the production of IGF1 in liver tissues and mediate growth promotion and metabolic regulation processes 7,8. This phenomenon predisposes individuals with LS to manifestations of hepatic steatosis 9. However, the mechanisms linking LS and hepatic steatosis remain poorly understood. Thus, it is crucial to improve our understanding of the pathophysiology of LS and its comorbidities.

The pathogenesis of LS induced by *GHR* mutation is currently explored through systematic or conditional knockout (KO) of *Ghr* in various tissues and organs of mice 10-15. However, the effect of *GHR* mutation on lipid metabolism differs between mice and humans. LS patients with increased serum levels of free fatty acids (FFAs) often develop fatty liver; however, mice carrying a global *Ghr* KO show normal serum levels of FFAs and do not develop hepatic steatosis. Therefore, this mouse model cannot fully simulate human LS and cannot be used to draw conclusions about the lipid metabolism in human LS 16. Minipigs, mammals with an adult weight of approximately 30 kilograms are small in size and easy to handle. Moreover, lipid metabolism in minipigs is more similar to that in humans, and fat digestion and absorption in minipigs are similar to those in humans 17,18. Additionally, changes in blood lipids in minipigs fed a high-fat diet are consistent with those observed in obese humans 19. Therefore, minipigs are more suitable than mice as for modelling human lipid metabolism-related diseases 20,21.

Here, we established a *GHR* KO pig model 22 to investigate the cause of abnormal lipid metabolism. Initially, we found that deletion of *GHR* in pigs resulted in an imbalance in glucose and lipid metabolism, culminating in marked hepatic steatosis. Transcriptome analysis showed significant changes in signals related to lipid metabolism. Subsequently, we used si*GHR*-transfected human hepatocytes to confirm that *GHR* deletion affected affect expression of the key fatty acid oxidation enzymes ACOX1 and CPT1A by reducing the expression of AHR, which triggered abnormal fatty acid degradation and led to fat deposition. Our findings revealed the important role of liver GHR in lipid metabolism.

## Materials and methods

### Animals

*GHR* KO pigs on the China Experimental Mini Pigs background were created using the zinc finger nuclease (ZFN) system. Pig genotyping was performed by PCR with the primers 5'-AAGCGGTGTCTATGTGCTGATTCTC-3' and 5'-TCAGTGGCTAGAGTATATGATGTTG-3'; the WT and targeted alleles produced PCR bands of 530 bp and 534 bp, respectively. The mRNA level of the *GHR* gene was confirmed by Q-PCR, and the protein level of GHR was evaluated by Western blotting and immunohistochemistry with an anti-GHR antibody (Bioss, China).

### Cell culture

HepG2 and L02 cells were maintained in our laboratory. These cells were maintained in Dulbecco's modified Eagle's medium (DMEM, Gibco, Grand Island, NY, USA) supplemented with 10% heat-inactivated foetal bovine serum (FBS) at 37 °C in a humidified atmosphere of 5% CO_2_.

Hepa1-6 cells were purchased from Procell and were maintained in DMEM supplemented with 10% heat-inactivated FBS, 1 mM sodium pyruvate and 1% penicillin/streptomycin (P/S) at 37 °C in a humidified atmosphere of 5% CO_2_.

### Plasmid construction

The promoter sequence of *ACOX1* was cloned into the pGL3-Basic vector to construct the pGL3-*ACOX1* plasmid. The pGL3-*CPT1A* was constructed similarly. The coding sequence (CDS) of *AHR* was cloned into the pCMV-Myc vector to construct pCMV-Myc*-AHR.*

### *GHR* siRNA treatment in hepatocytes

*GHR* knockdown cells were established by using an siRNA directed against *GHR*. HepG2, L02 and Hepa1-6 cells were transfected with si*GHR* using Lipofectamine 2000 transfection reagent (Life Technologies, Grand Island, NY) in accordance with the manufacturer's instructions. First, cells were cultured in 12-well plates overnight. Then, 40 pmol negative control (NC) siRNA or si*GHR* mixed with Lipofectamine was added to each well. The cells were collected after 24 hours for follow-up experiments.

### *AHR* siRNA treatment in hepatocytes

*AHR* knockdown cells were established by using an siRNA directed against *AHR*. HepG2 were transfected with si*AHR* using Lipofectamine 2000 transfection reagent (Life Technologies, Grand Island, NY) in accordance with the manufacturer's instructions. First, cells were cultured in 12-well plates overnight. Then, 40 pmol negative control (NC) siRNA or si*AHR* mixed with Lipofectamine was added to each well. The cells were collected after 24 hours for follow-up experiments.

### Analysis of plasma metabolites

Plasma glucose, insulin, TGs, TC, HDL, LDL, FFAs, ALT and AST were measured by Beijing Zhongtong Lanbo Clinical Laboratory.

### Intravenous glucose tolerance test (IGTT)

After fasting blood glucose levels were measured, each pig was injected intravenously with 1.2 ml of a 50% glucose solution per kilogram body weight (ml kg^-1^). Blood glucose levels were then measured at 0, 2, 5, 10, 20, 30, 60 and 120 minutes post injection using a glucometer.

### Measurement of the TG content in liver tissue and cells

Livers collected from the pigs were stored at -80 °C until use. The TG content in the liver and hepatocytes was determined using a Triglyceride Assay Kit according to the manufacturer's protocol (Nanjing Jiancheng Bioengineering Institute, China). The protein concentration was determined with a BCA Protein Assay Kit (Beyotime Biotechnology, China).

### Q-PCR

Total RNA was extracted with TRIzol (Invitrogen, USA), and reverse transcription was performed with 1 μg of total RNA using PrimeScript™ RT Master Mix (TaKaRa, Japan). SYBR Green PCR Master Mix (Roche, Switzerland) was used for PCR amplification. All reactions were performed in triplicate. The mRNA expression levels of the target genes were normalized to that of *GAPDH*. The primers used are listed in Supplementary Table S1.

### Western blot analysis

Total cell lysates were prepared from either liver tissue or cultured cells by lysis using RIPA Lysis Buffer (Beyotime, China) containing 1% PMSF and homogenization using a vortex oscillator (Roche, USA). Protein concentrations were determined using an Enhanced BCA Protein Assay Kit (Beyotime, China). An equal volume of 5 X loading buffer was mixed with the samples, which were boiled for 10 minutes. After separation by 10% SDS-PAGE, the proteins were transferred to a 0.45 mm PVDF membrane and blocked for 2 hours with 5% non-fat dry milk at room temperature. Then, the membrane was incubated overnight at 4 °C with the appropriate primary antibody. After incubation with the secondary antibody for another hour, the membranes were developed with SuperSignal™ West Pico PLUS Chemiluminescent Substrate (Thermo, USA). The following primary antibodies were used. Antibodies against ACOX1 (A8091) and CPT1A (A5307) were purchased from ABclonal, and an antibody against AHR (#4685) was purchased from Cell Signaling Technology (Danvers, MA, USA). The antibody against GAPDH (AG019) was purchased from Beyotime Biotechnology.

### Histopathological, IHC and immunofluorescence (IF) staining

Tissues were fixed with 4% paraformaldehyde and then sequentially dehydrated in increasing concentrations of ethanol ranging from 75 to 100%. Dehydrated specimens were subsequently cleared with xylene, embedded in paraffin, processed for paraffin sectioning (5-mm sections), and subjected to H&E, IHC or IF staining. For IHC staining, sections were incubated overnight with primary antibodies and then with HRP-conjugated secondary antibodies. For IF staining, the sections were incubated overnight with primary antibodies and then with Alexa Fluor® 594/488-conjugated secondary antibodies and DAPI (Sigma).

### Oil red O staining

Samples of liver were embedded in O.C.T. compound (Solarbio China). Fresh frozen specimens were cryosectioned at a thickness of 6 μm and air dried. The sections were then fixed in PBS for 5 minutes and, after soaking for 5 minutes in 60% isopropanol, stained with a 3:2 mixture of 0.5% Oil red O solution in isopropanol and distilled water for 15 minutes in the dark. The slides were washed with 60% isopropanol for 10 s and then washed with distilled water. Nuclei were then stained with haematoxylin for 5 minutes, bluing was performed with tap water, and the sections were sealed with glycerol-gelatine and observed under a microscope.

### Nile red staining

Nile red staining was used for cellular lipid droplet staining. The cells to be tested were washed twice with PBS for 3 minutes each, fixed with 4% paraformaldehyde for 20 minutes, washed twice with PBS for 3 minutes each, stained with Nile red at 37 °C for 20 minutes, and then washed with PBS 2 times for 3 minutes each. DAPI was used to stain the nuclei for 20 minutes, and the cells were finally washed twice with PBS and photographed under a microscope.

### Luciferase reporter assay

L02 cells were plated in a 24-well culture plate and transfected with a reporter vector (500 ng) together with each indicated expression plasmid using Lipofectamine 2000 (Invitrogen) according to the manufacturer's instructions. Luciferase activities were measured using a Dual Luciferase Reporter Assay System (Promega, Madison, WI, USA) according to the manufacturer's instructions. Firefly luciferase activity was normalized to Renilla luciferase activity.

### Chip assay

L02 cells were plated in a 10 cm^2^ culture plate. After incubation with the AHR agonist Tapinarof (Topscience, T4644), for 12 h, the cells were collected, and a ChIP assay was performed using a SimpleChIP Enzymatic Chromatin Immunoprecipitation Kit (Cell Signaling Technology, #9003) according to the manufacturer's protocol. The primers used for real-time PCR analysis of the DNA fragments of interest among the immunoprecipitated DNA fragments are listed in Supporting Table S.

### CO-IP

Pig livers and human hepatocytes were used to study whether GHR and AHR have a related effect. To the human hepatocytes was added AHR ligand Tapinarof. The cells were harvested after 24 hours, and the IP experiment was conducted according to Beyotime's kit. The final product was verified by Western blot analysis.

### Statistical analysis

Data are expressed as the means ± SDs and were analysed using GraphPad Prism Software 7.00. For comparisons between two groups of data that showed normal distribution and homogeneity of variance, the two-tailed Student's t-test was performed. P-values <0.05 were considered statistically significant.

## Results

### Knockout of *GHR* in pig results in a severe dwarf phenotype

**Figure 1:**
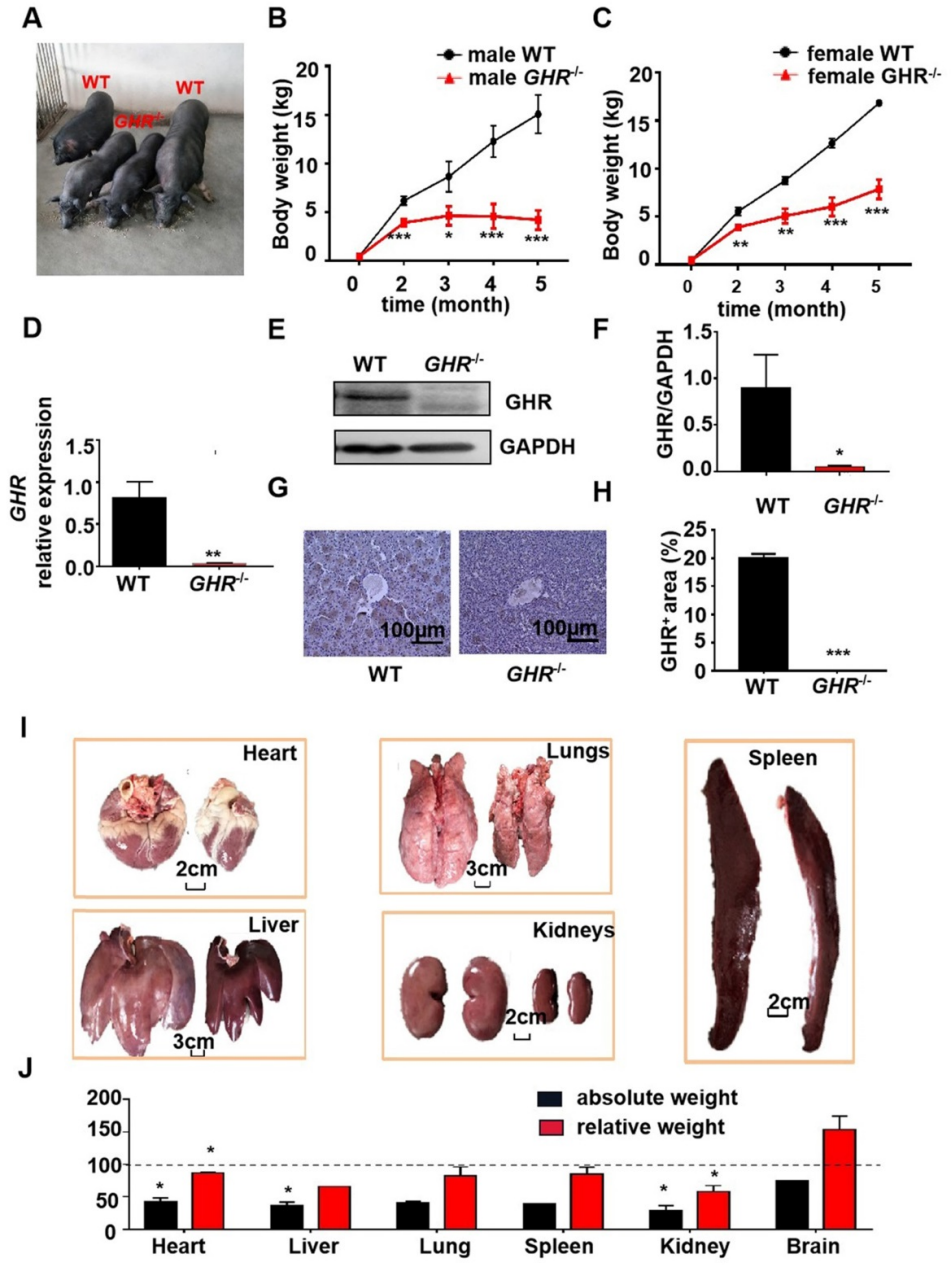
** Phenotype of *GHR* KO pigs. (A)** Representative photographs of pigs of the two genotypes. **(B-C)** Body weight of male and female* GHR* KO pigs and their WT littermates. **(D-F)** Hepatic *GHR* mRNA and protein levels in *GHR* KO pigs. (D) Hepatic *GHR* mRNA levels in *GHR* KO pigs. (E-F) Hepatic GHR protein levels in *GHR* KO pigs by Western blotting, and quantification of the content by ImageJ. **(G-H)** Hepatic GHR protein levels in *GHR* KO pigs by IHC and quantification of the content by ImageJ. **(I)** Representative photographs of organs from *GHR* KO pigs and their WT littermates. **(J)** Absolute weight and relative weight of the organs. Scale bar: 100 µm. n = 3 pigs per group. The data are presented as the mean ± SD values. *, P < 0.05; **, P < 0.01; ***, P < 0.001. Annotation: relative weight, the ratio of organ weight to body weight.

To determine the physiological roles of GHR *in vivo*, we generated *GHR* KO pigs (Figure 1A). The mRNA expression of *GHR* was markedly reduced in *GHR* KO pigs compared with wild-type (WT) pigs (Figure 1D), and the protein expression of GHR was downregulated in liver samples from *GHR* KO pigs (Figure 1E-F). In addition, immunohistochemical (IHC) staining of liver sections further indicated that the expression of GHR was decreased in the livers of *GHR* KO pigs (Figure 1G-H). We measured the growth rate of *GHR* KO pigs and observed that the weights of both male and female GHR KO pigs were approximately half those of WT pigs (Figure 1B-C), and the lengths were also significantly reduced (Supplementary Figure 1A-B). However, heterozygous *GHR* mutant pigs did not show any significant difference in GHR protein level, body weightor body length (Supplementary Figure 1C-F). Consistent with their smaller size,* GHR* KO pigs had a lower absolute weight of all organs than WT pigs. After normalization to the whole body weight, the heart, liver, spleen, lung, and kidney also showed significantly reduced relative weights, but the relative brain weights were increased in *GHR* KO pigs (Figure 1G-H).

### *GHR* deficiency disrupts glucose/lipid homeostasis in pigs

**Figure 2:**
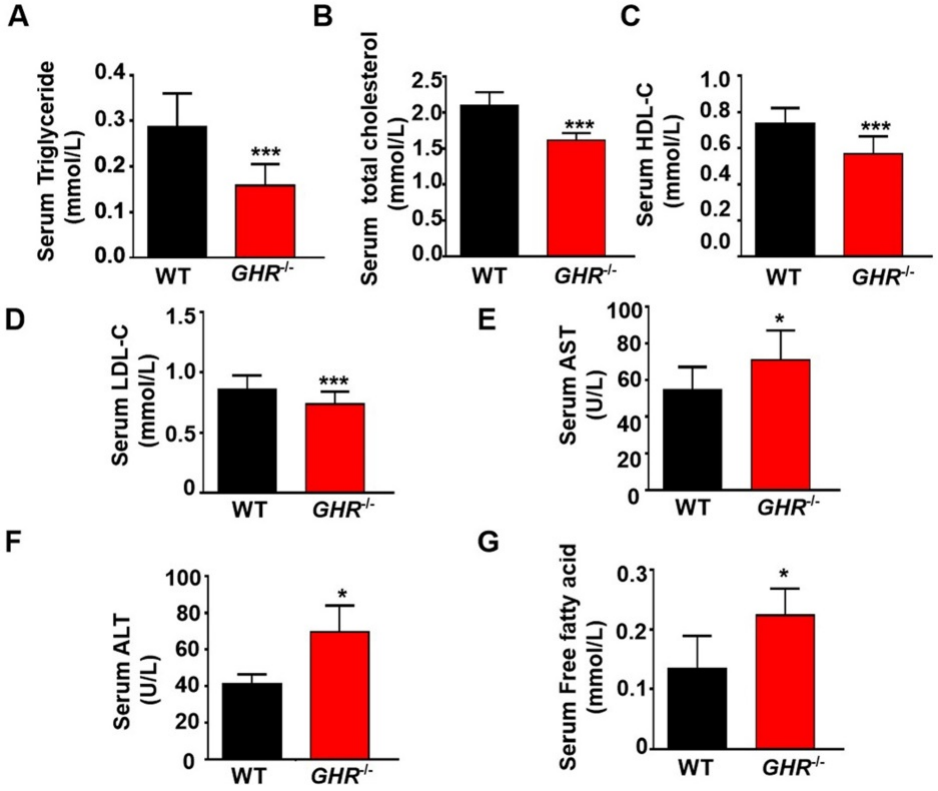
**GHR deficiency disrupted lipid homeostasis in pigs. (A-E)** Serum TG, TC, HDL, LDL and FFA levels in WT and GHR KO pigs. **(F-G)** Liver function markers, including ALT and AST, in WT and GHR KO pigs. n = 3 pigs per group. The data are presented as the mean ± SD values. *, P < 0.05; **, P < 0.01; ***, P < 0.001.

GH can increase hepatic glucose production 23, and GH-GHR signaling plays a major role in regulating glucose metabolism 24. Thus, we investigated the effect of GHR deficiency on glucose metabolism and observed that fasting blood glucose was significantly decreased in *GHR* KO pigs (Supplementary Figure 2A). In addition, the serum insulin level and homeostatic model assessment of insulin resistance (HOMA-IR) index were significantly lower in *GHR* KO pigs (Supplementary Figure 2B-C). The homeostatic model assessment of insulin sensitivity (HOMA-IS) index was significantly higher in *GHR* KO pigs (Supplementary Figure 2D). When glucose was administered by i.p. injection, *GHR* KO pigs exhibited glucose intolerance (Supplementary Figure 2E-F). PI3K-AKT is the main downstream signaling pathway of insulin. Activated AKT increases glycogen synthesis and inhibits gluconeogenesis, thereby reducing blood sugar and increasing insulin sensitivity 25. Therefore, Western blotting was used to detect AKT molecules. The results showed that the phosphorylation of AKT was significantly increased in *GHR* KO pigs (Supplementary Figure 2G-H), which could explain the insulin sensitivity symptoms of *GHR* KO pigs. Similarly, we used *GHR* KO pigs to confirm the function of GHR in lipid metabolism. Biochemical analysis showed large decreases in triglycerides (TGs), total cholesterol (TC), high-density lipoprotein (HDL) and low-density lipoprotein (LDL) in *GHR* KO pigs (Figure 2A-F). Notably, the serum FFA level in *GHR* KO pigs was significantly increased, consistent with the results in LS patients (Figure 2G). In summary, these findings indicate that disruption of *GHR* has a major effect on glucose and lipid homeostasis and that the resulting phenotype is more similar to that observed in human patients.

### *GHR* deficiency induces hepatic steatosis in pigs

**Figure 3:**
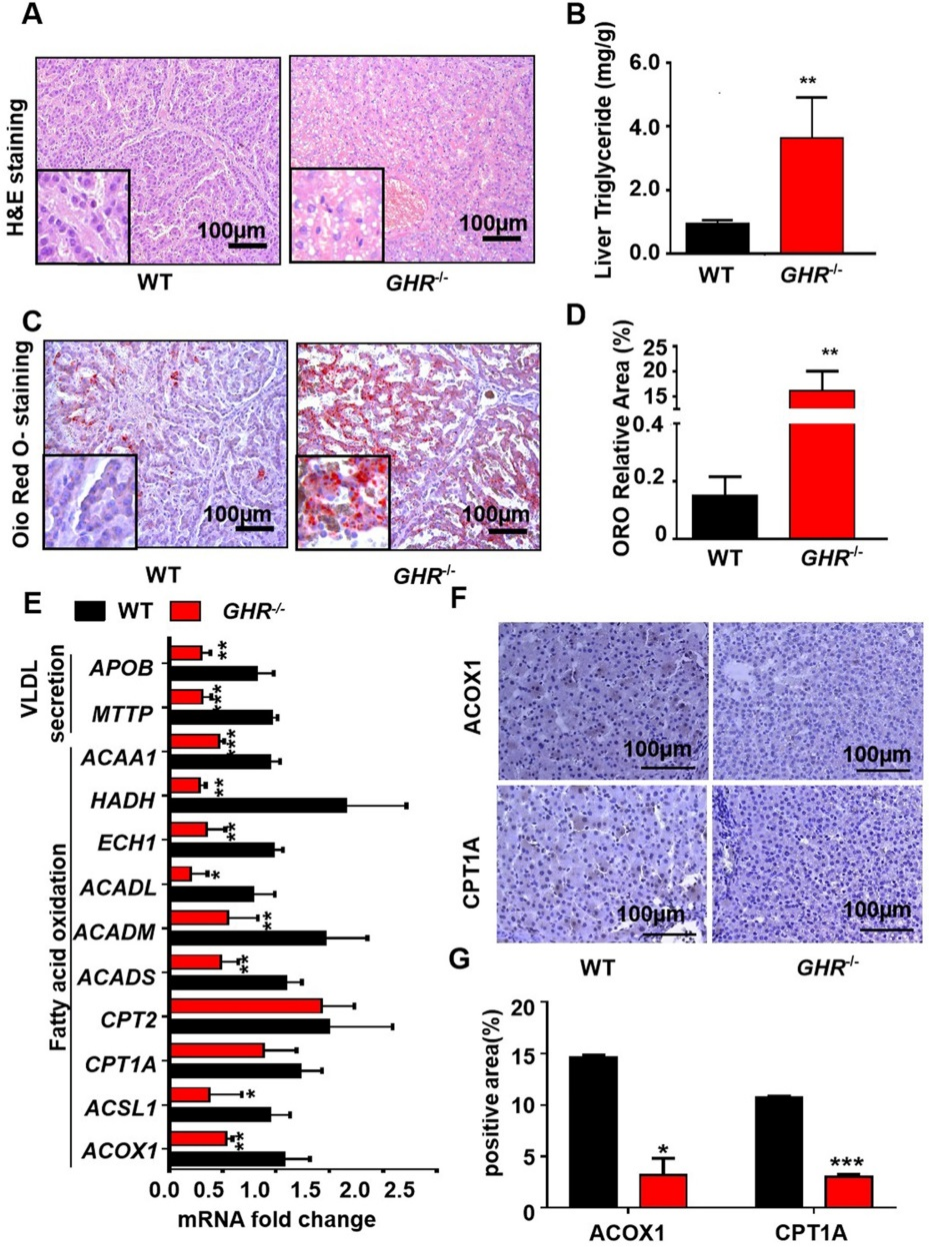
***GHR* KO pigs developed hepatic steatosis. (A)** Representative image of H&E staining. **(B)** Hepatic TG levels in WT and *GHR* KO pigs. **(C-D)** Representative image of Oil red O staining and relative Oil red O-stained area. **(E)** mRNA levels of key genes in fatty acid oxidation and VLDL secretion. **(F-G)** Protein levels of key genes in fatty acid oxidation and quantification of the content by ImageJ. Scale bar: 100 µm. n = 3 pigs per group. The data are presented as the mean ± SD values. *, P < 0.05; **, P < 0.01; ***, P < 0.001.

Owing to the critical role of GHR in lipid metabolism, we focused mainly on whether GHR deficiency affects hepatic steatosis in pigs. Biochemical analysis confirmed a significant increase in TGs in the livers of *GHR* KO pigs (Figure 3B), accompanied by increases in alanine aminotransferase (ALT) and aspartate aminotransferase (AST) suggestive of liver damage (Figure 2E-F). Accordingly, histological analyses of livers based on haematoxylin and eosin (H&E) staining revealed increased numbers and sizes of intracellular vacuoles (an indication of increased fat deposition) in *GHR* KO pigs (Figure 3A). Oil red O (ORO) staining of liver sections verified the deposition of increasing quantities of lipids in *GHR* KO pigs (Figure 3C-D). The above results suggest that GHR deficiency leads to hepatic steatosis in pigs.

### *GHR* deficiency reduces fatty acid oxidation in pigs

A series of studies was conducted to identify the source of hepatic steatosis in *GHR* KO pigs. The mRNA expression of genes responsible for fatty acid oxidation was significantly downregulated in *GHR* KO pigs (Figure 3E). In particular, ACOX1 and CPT1A are the two rate-limiting enzymes of fatty acid oxidation 26. The IHC results showed that the protein levels of ACOX1 and CPT1A were lower in *GHR* KO pigs (Figure 3F-G). Moreover, MTTP and APOB, two important indicators of VLDL secretion, were also significantly downregulated in GHRKO pigs. (Figure 3E), although the mRNA levels of fatty acid uptake-, synthesis- and inflammation- related genes were not significantly different (Supplementary Figure 3A). Taken together, these lines of evidence suggest that *GHR* deletion results in hepatic steatosis. Notably, *GHR* KO led not only to lipid accumulation in the pig liver but also to significant accumulation of lipids in the kidney, skeletal muscle and pancreas. Additionally, the size of adipocytes in *GHR* KO pigs increased significantly (Supplementary Figure 4).

### Knockdown of *GHR* causes intracellular lipid accumulation in cultured human hepatocytes

**Figure 4:**
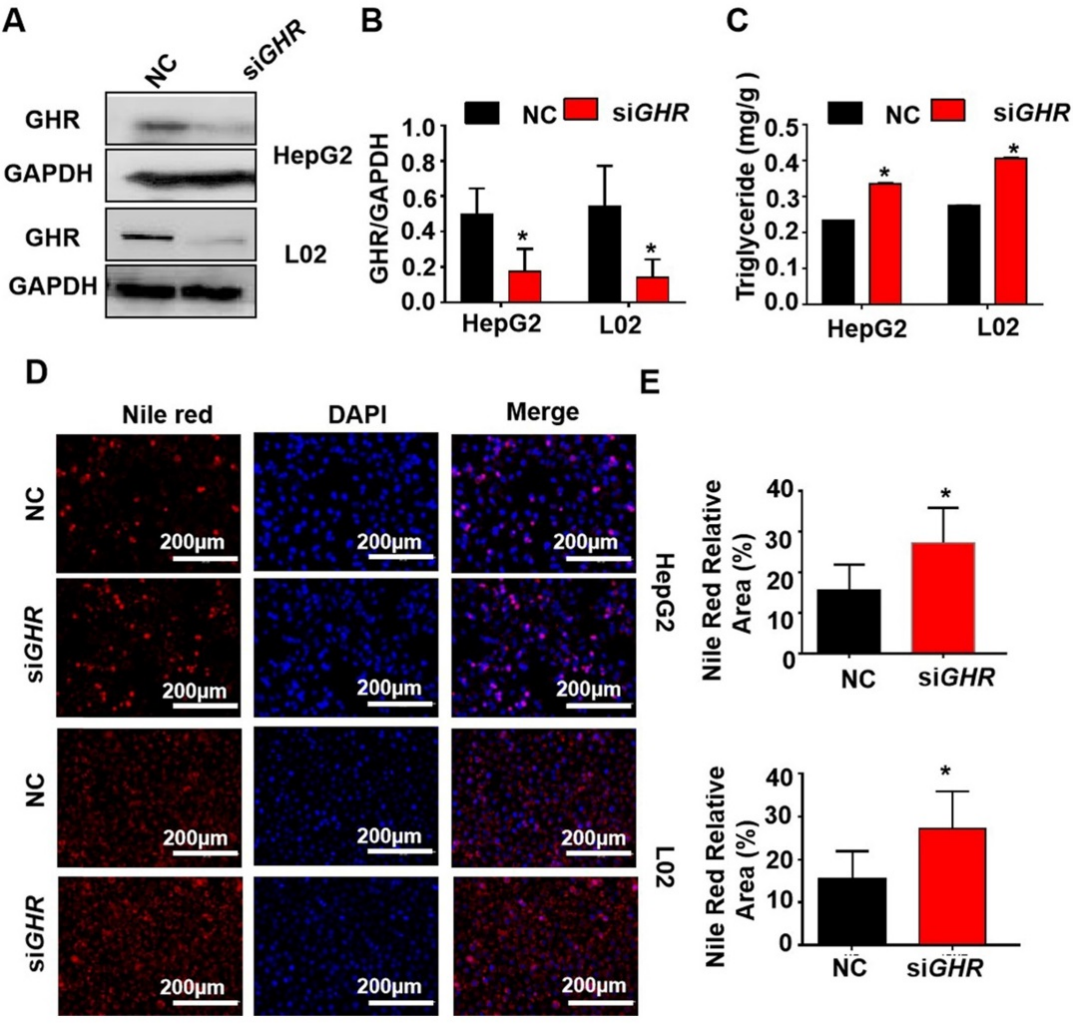
***GHR* depletion caused intracellular lipid accumulation in cultured human hepatocytes. (A-E)** Hepatocytes were treated with NC or *GHR* siRNA (si*GHR*). (A-B) The GHR protein expression levels in human hepatocytes, and quantification of the content by ImageJ. (C) TG levels in human hepatocytes. (D) Nile red staining of human hepatocytes with NC or si*GHR*. (E) The neutral lipid content was quantified with ImageJ and normalized to the number of nuclei. Scale bar: 100 µm. n = 3 per group. The data are presented as the mean ± SD values. *, P < 0.05; **, P < 0.01; ***, P < 0.001.

To explore whether hepatic steatosis caused by *GHR* KO in pigs also occurs in humans, si*GHR* was transfected into two human hepatocyte lines, HepG2 and L02, to silence *GHR*. Western blot analysis showed that GHR expression was markedly decreased in si*GHR*-treated human hepatocytes (Figure 4A). The mRNA levels of genes responsible for fatty acid oxidation were significantly downregulated in si*GHR*-treated human hepatocytes (Supplementary Figure 3B). Consistent with the results in *GHR* KO pigs, no significant difference was detected in the mRNA levels of genes related to fatty acid transport and synthesis in si*GHR*-treated human hepatocytes (Supplementary Figure 3B). The level of intracellular TGs was increased in si*GHR* human hepatocytes (Figure 4C), and Nile red staining indicated that lipid deposition was increased in si*GHR* human hepatocytes (Figure 4D-F). As a comparison, we also conducted similar experiments on si*Ghr* mouse hepatocytes. Quantitative real-time- PCR (Q-PCR) showed that *Ghr* expression was markedly decreased in si*Ghr* Hepa1-6 compared with control cells (Supplementary Figure 5A). Unlike the results in humans and pigs, there were no significant changes in the mRNA levels of genes related to fatty acid oxidation in si*Ghr* Hepa1-6 cells (Supplementary Figure 3C). Moreover, the levels of intracellular TGs (Supplementary Figure 5B) and Nile red staining (Supplementary Figure 5C-D) were not significantly different in si*Ghr* mouse hepatocytes. These results indicate that knockdown of *GHR* leads to intracellular lipid accumulation in human hepatocytes but not in mice. Taken together, we conclude that *GHR* KO pigs more accurately mimic abnormal lipid metabolism in LS patients.

### Transcriptional profile of *GHR* KO pigs during hepatic steatosis

**Figure 5:**
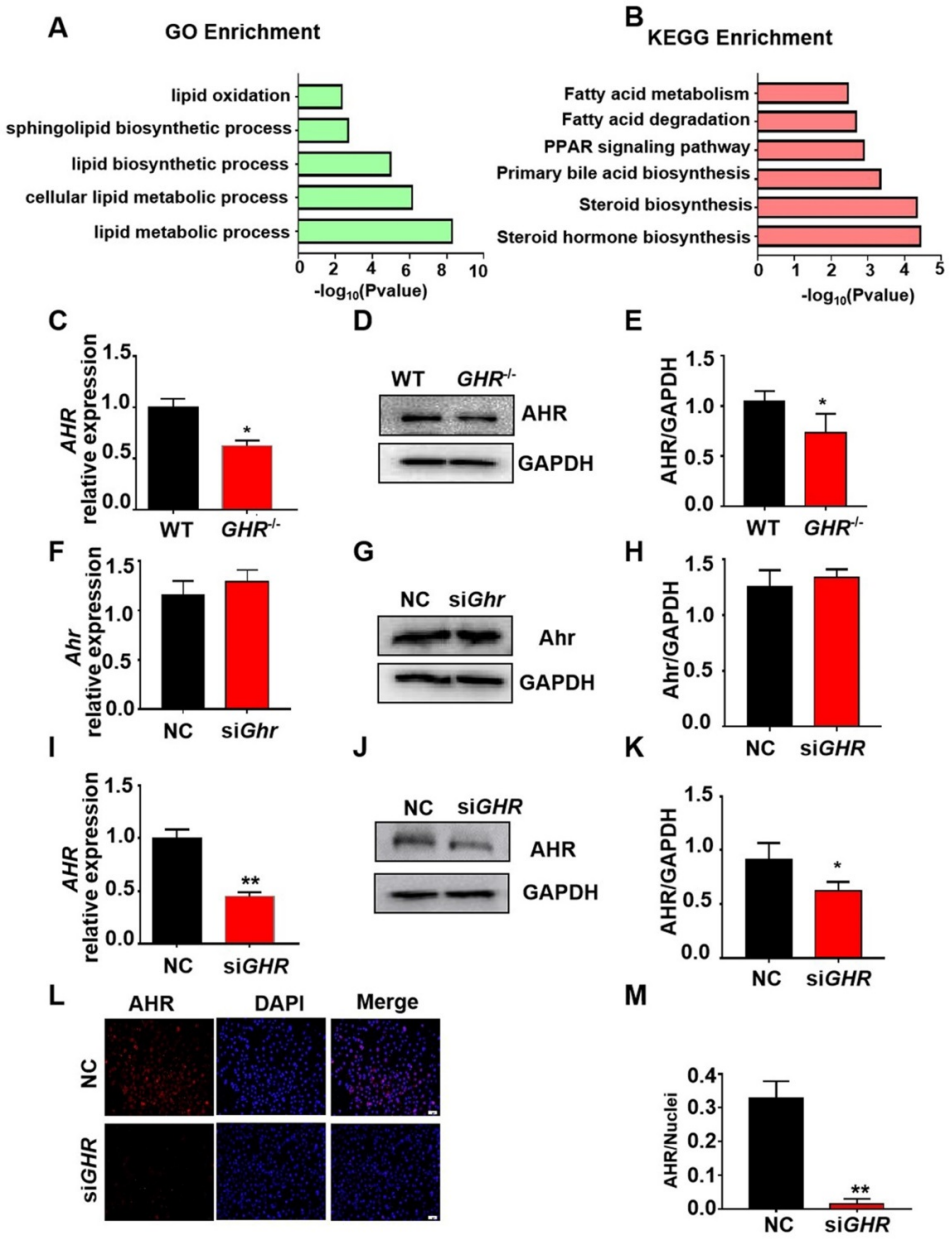
**Transcriptome analysis of hepatic gene expression profiles in *GHR* KO pigs. (A)** The five enriched biological processes contributing to *GHR* function were determined by GO analysis based on the DEGs. **(B)** KEGG pathway enrichment analysis of the six identified lipid metabolism-related processes. **(C-E)** mRNA and protein levels of AHR in the livers of WT and *GHR* KO pigs. **(F-H)** mRNA and protein levels of AHR in NC and si*Ghr* mouse hepatocytes. **(I-M)** mRNA and protein levels of AHR in NC and si*GHR* human hepatocytes. **(I)** mRNA levels of AHR in NC and si*GHR* human hepatocytes. **(J-K)** Protein levels of AHR in NC and si*GHR* human hepatocytes by Western blotting and quantification of the content by ImageJ. **(L-M)** Protein levels of AHR in NC and si*GHR* human hepatocytes by immunofluorescence and quantification of the content by ImageJ. Scale bar: 100 µm. n = 3 per group. The data are presented as the mean ± SD values. *, P < 0.05; **, P < 0.01; ***, P < 0.001.

To explore the causes of hepatic steatosis resulting from *GHR* KO, we performed RNAseq analysis on liver tissue from *GHR* KO pigs. With the cut-off criteria of a false discovery rate < 0.05 and Fold Change ≥2, 897 (306 upregulated, 591 downregulated) differentially expressed genes (DEGs) were identified in *GHR* KO pigs (Supplementary Figure 6A-B). Gene ontology (GO) term and Kyoto Encyclopedia of Genes and Genomes (KEGG) pathway analyses were performed with the lipid metabolism-related genes. GO terms (Figure 5A) related to lipid oxidation, sphingolipid biosynthetic process, and lipid metabolic process and KEGG pathways (Figure 5B) of fatty acid metabolism and fatty acid degradation were enriched. The transcriptome data also identified some differentially expressed transcription factors (TFs), among which AHR, an important transcription factor in the regulation of lipid metabolism, was significantly downregulated in GHR KO pigs (Supplementary Figure 6C). The mRNA and protein levels of AHR were significantly reduced in *GHR* KO pigs and si*GHR* human hepatocytes (Figure 5C-E, I-M), whereas the expression of Ahr was not changed in si*Ghr* mouse hepatocytes (Figure 5F-H). These results suggest that AHR misregulation may be responsible for lipid accumulation in humans and pigs.

### GHR regulates AHR expression through the MAPK/ERK signaling pathway

**Figure 6:**
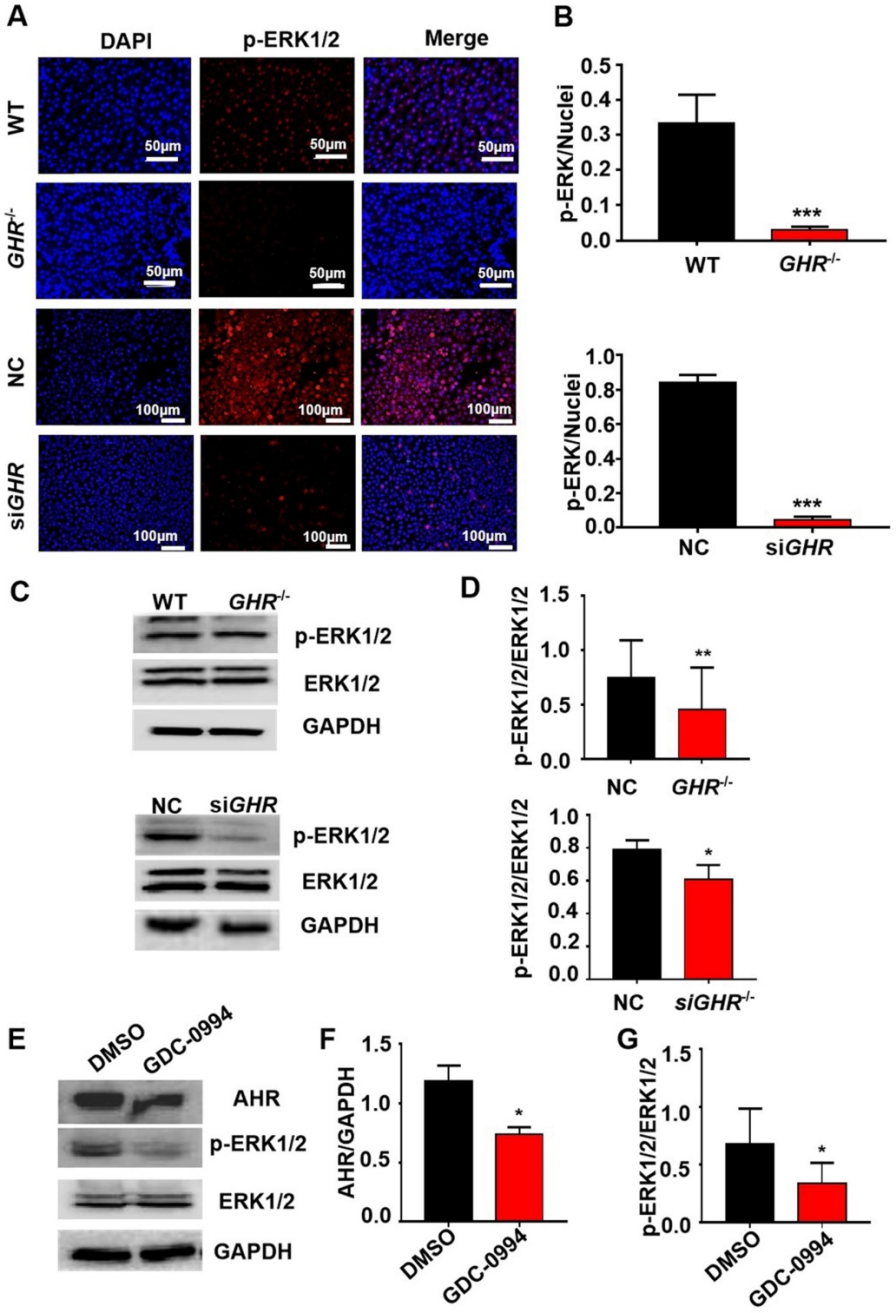
** GHR regulates AHR expression through the MAPK signaling pathway. (A-B)** IF staining of p-ERK1/2 in pigs and hepatocytes, and quantification of the content by ImageJ. **(C-D)** Protein levels of p-ERK1/2 and ERK1/2 in pigs and hepatocytes by Western blotting and quantification of the content by ImageJ. **(E-G)** Protein levels of AHR, p-ERK1/2 and ERK1/2 in L02 cells treated with GDC-0994 (an ERK inhibitor), and quantification of the content by ImageJ. Scale bar = 50 µm. n = 3 per group. The data are presented as the mean ± SD values. *, P < 0.05; **, P < 0.01; ***, P < 0.001.

**Figure 7:**
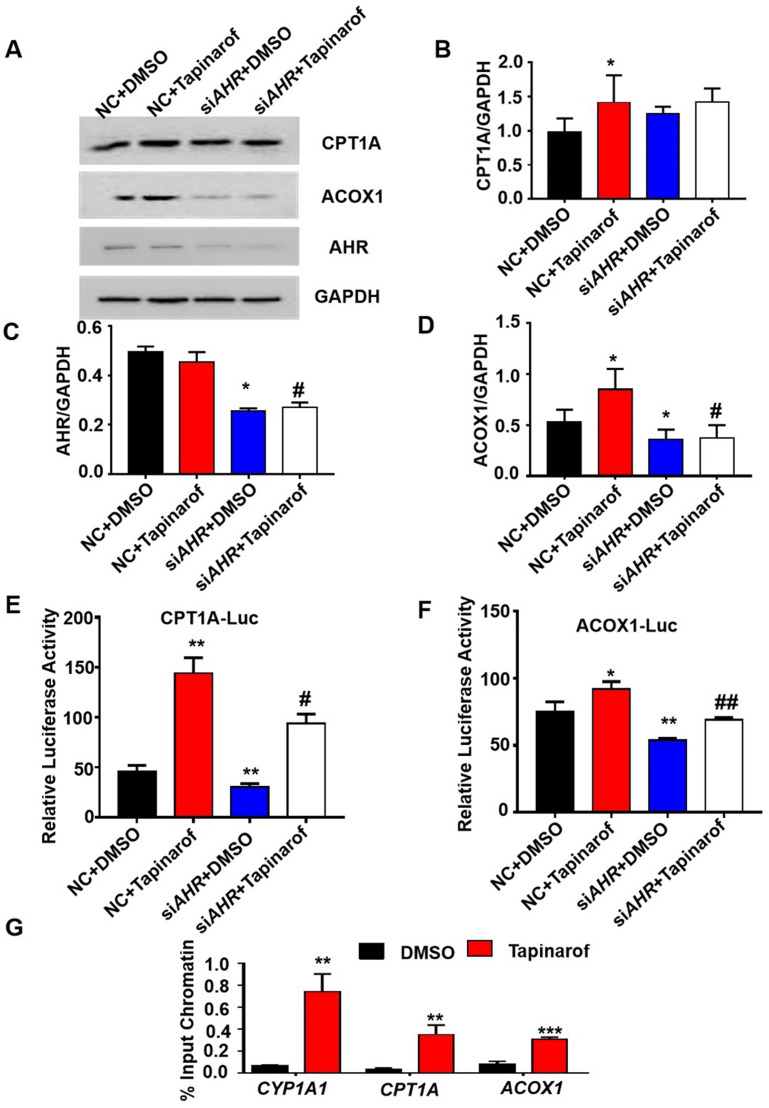
** AHR directly activated fatty acid oxidation gene expression. (A-D)** Protein levels of CPT1A, ACOX1 and AHR in cells treated with Tapinarof (an AHR ligand) and/or *AHR* siRNA (si*AHR*), and quantification of the content by ImageJ. **(E)** Effect of Tapinarof and/or si*AHR* treatment on *ACOX1* luciferase reporter activity. **(F)** Effect of Tapinarof and/or si*AHR* treatment on *CPT1A* luciferase reporter activity. **(G)** ChIP assays were performed with the *CYP1A1*, *CPT1A and ACOX1* promoters in L02 cells with or without Tapinarof (10 µM) treatment for 12 h. n = 3 per group. The data are presented as the mean ± SD values. *, P < 0.05; **, P < 0.01; ***, P < 0.001 compared with the first group, ^#^, P < 0.05; ^##^, P < 0.01; ^###^, P < 0.001 compared with the second group.

The above results show that by interfering with the expression of GHR, AHR is significantly downregulated. To explore the mechanism of AHR involvement in hepatic steatosis caused by GHR deletion, we used Co-IP experiments to verify whether there is a protein interaction between AHR and GHR. The results showed that AHR did not directly interact with GHR (Supplementary Figure 7A-B). Given that MAPK is the classical signaling pathway downstream of GHR and the confirmed crosstalk between the MAPK and AHR pathways, we speculated that GHR may influence the expression of AHR through MAPK. First, immunofluorescence results showed that the phosphorylation levels of ERK1/2 were significantly decreased in both *GHR* KO pigs and si*GHR* human hepatocytes (Figure 7A-B). Western blot findings confirmed this conclusion (Figure 6C-D). Furthermore, Western blot analysis confirmed that blocking the function of ERK1/2 with GDC-0994 resulted in the downregulation of AHR expression (Figure 6E-G). These results suggest that GHR regulates the expression of AHR through the MAPK/ERK signaling pathway.

### AHR directly activates fatty acid oxidation gene expression

**Figure 8:**
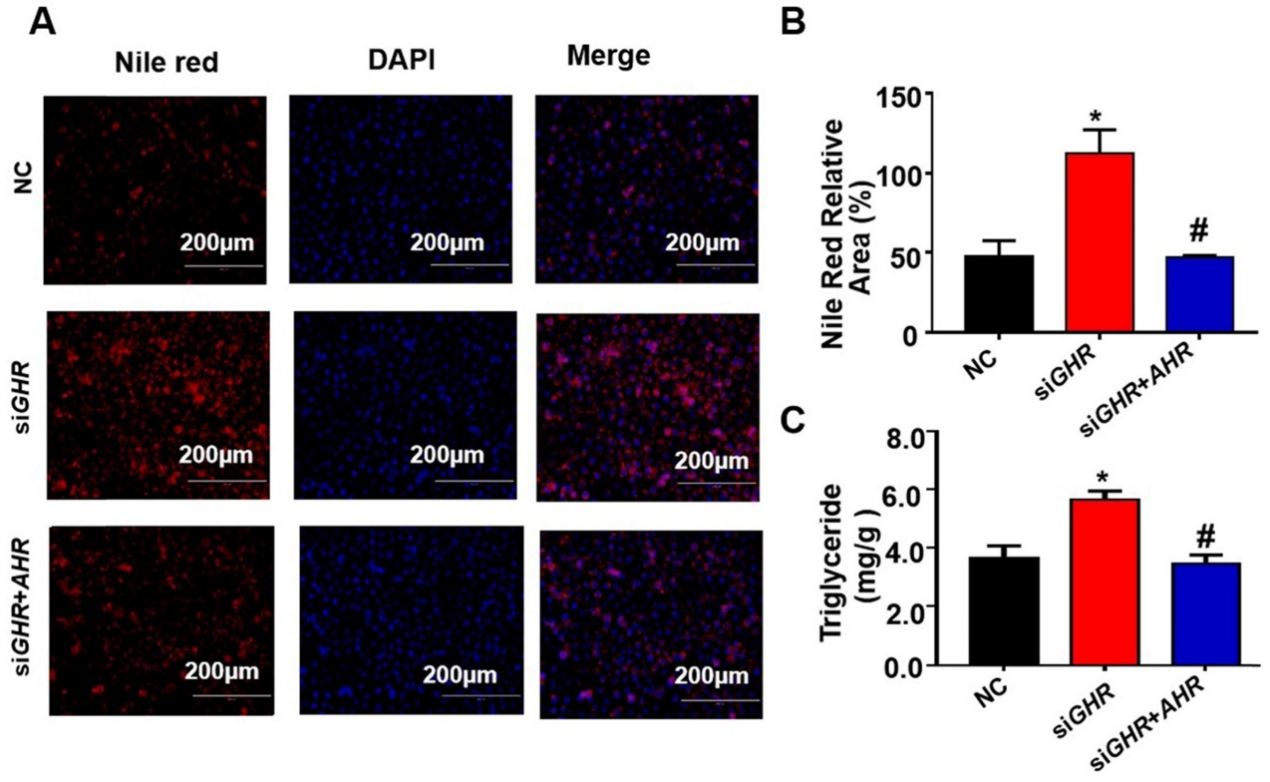
**Overexpression of AHR alleviated lipid deposition induced by *GHR* deletion. (A)** Nile red staining and (**C**) TG content in NC and si*GHR* cells with or without pCMV-Myc*-AHR* transfection. **(B)** The neutral lipid content was quantified with ImageJ and normalized to the number of nuclei. The data are presented as the mean ± SD values. Scale bar: 100 µm. n = 3 per group. *, P < 0.05; **, P < 0.01; ***, P < 0.001 compared with the first group; ^#^, P < 0.05; ^##^, P < 0.01; ^###^, P < 0.001 compared with the second group.

To investigate the relationship between the expression of AHR and fatty acid oxidation in hepatic steatosis induced by *GHR* deletion, we examined the expression of genes related to fatty acid oxidation. The protein levels of ACOX1 and CPT1A in human hepatocytes were increased after the addition of Tapinarof (an AHR ligand), while, AHR knockdown significantly decreased Tapinarof-induced ACOX1 and CPT1A expression at protein level (Figure 7A-B). To assess whether ACOX1 and CPT1A are directly regulated by AHR, we checked their activity in hepatocytes following Tapinarof treatment. The luciferase reporter assay manifested that stimulating the AHR with Tapinarof promoted the activity of ACOX1 and CPT1A, whereas AHR knockdown by siAHR treatment had the opposite effect (Figure 7C-D). Next, chromatin immunoprecipitation (ChIP) assays and real-time PCR analysis of the immunoprecipitated DNA demonstrated that AHR directly bound to the promoter regions of CPT1A and ACOX1 (Figure 7E). The above results indicate that AHR directly activates the expression of fatty acid oxidation genes at the transcriptional level. To explore whether the protective effects of *GHR* are dependent on AHR in hepatic steatosis, we carried out the rescue experiments on si*GHR* human hepatocytes. The results of TG measurement and Nile red staining showed that overexpression of AHR could alleviate lipid deposition induced by *GHR* deletion (Figure 8A-B).

## Discussion

Here we report a large animal model of LS caused by *GHR* deficiency. Most previous studies have used the *Ghr* KO mouse model to explore the function of GH and have provided new insights into the consequences of GH insensitivity for metabolic functions 27. *Ghr* KO mice showed increased serum levels of GH and decreased serum levels of IGF-I, obesity and hypoglycaemia 13,27,28. However, the influence of *GHR* mutation on lipid metabolism differs between mice and humans. The serum levels of FFAs in *Ghr* KO mice are reduced, while individuals with LS usually show increased FFA levels 27. Compared with mice, minipigs are similar to humans in terms of lipid metabolism 18,19. Therefore, we used gene editing technology to successfully knock out the *GHR* gene in pigs and establish the *GHR* KO model of LS; these animals showed typical dwarfism symptoms and obesity 22. Interestingly, unlike the reduced level of FFAs in *Ghr* KO mice and *GHR* KO pigs 27,29, the serum level of FFAs in our *GHR* KO pigs was significantly increased, which was consistent with the higher serum level of FFAs in LS patients 30. Therefore, our *GHR* KO pigs could more accurately mimic lipid metabolism in human LS.

As the central organ in metabolism, the liver participates in a variety of lipid metabolic pathways. Fatty liver begins with the deposition of TGs in hepatocytes and the formation of lipid droplets of different sizes. The increases in fat decomposition, the serum level of FFAs and fat synthesis and the decrease in fatty acid oxidation result in hepatic lipid accumulation 31-34. Patients with LS often tend to develop fatty livers 9. Interestingly, previous studies have found no hepatic steatosis in global *Ghr* KO mice 11, except liver-specific *Ghr* KO mice 35. However, our new observations revealed an important role of *GHR* deficiency in promoting hepatic steatosis. In our study, both *GHR* KO pigs and si*GHR* human hepatocytes manifested lipid accumulation and reduced fatty acid oxidation, which was not observed in si*Ghr* mouse hepatocytes. Therefore, reduced fatty acid oxidation may be an important cause of hepatic steatosis due to *GHR* deficiency. The above results further indicate that pigs can better simulate the symptoms of abnormal lipid metabolism in humans.

Inflammation is an important indicator of the development of liver steatosis to liver fibrosis 36. Unfortunately, due to the limitation of experimental conditions, we did not obtain experimental pigs of greater monthly age. Therefore, the current inflammation-related indicators of GHRKO pigs have not changed significantly, follow-up experiments are needed to explore in future.

Based on the analysis of our transcriptome data, 23 transcription factors were identified, and the transcription factor AHR was identified as one of the top hits, which has been linked to hepatic steatosis in previous reports. AHR can affect hepatic glucose and lipid metabolism in either a detrimental or beneficial manner 37,38. Some studies have shown that *CD36*, a key gene in fatty acid transport 39, is a new transcription target for AHR. Activation of AHR can induce *CD36* expression, enhance fatty acid uptake, and lead to hepatic steatosis 40. However, others have found that treatment with AHR agonists can reduce the synthesis of fatty acids 41. While the mRNA and protein levels of Ahr in si*GHR* mouse hepatocytes were normal, our results showed that the mRNA and protein levels of AHR in *GHR* KO pigs were reduced, and knockdown of *GHR* in human hepatocytes led to a significant downregulation of AHR, resulting in lipid accumulation, and that overexpression of *AHR* ameliorated lipid accumulation. Furthermore, we found that AHR could bind directly to the promoter regions of *ACOX1* and *CPT1A* (the key genes in fatty acid oxidation) and transcriptionally activate their expression.

**Figure 9:**
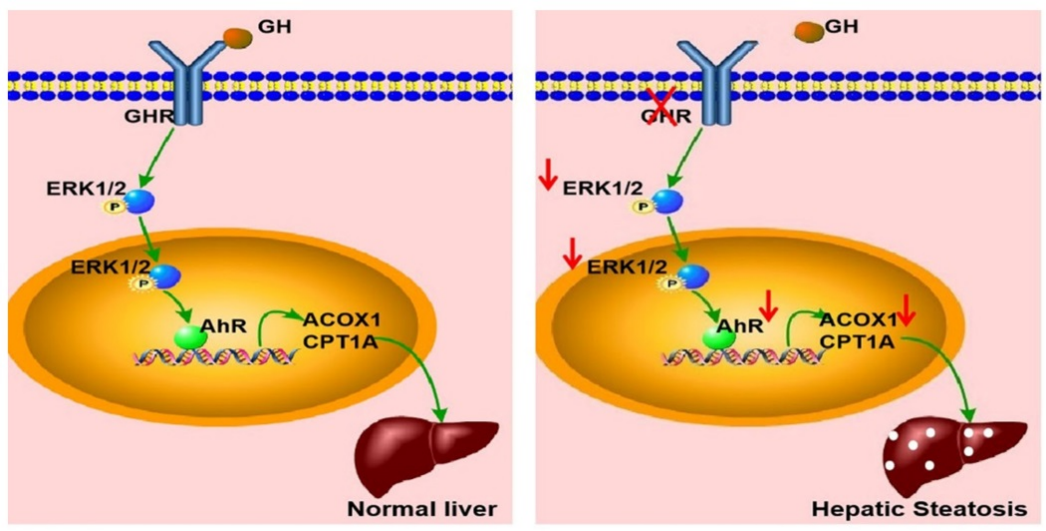
** Schematic illustration of the proposed role of GHR in hepatic steatosis.** Normal liver (left): GH binds to GHR and promotes the expression of AHR by activating ERK1/2 via phosphorylation. Activated AHR directly binds to the promoter regions of *ACOX1* and* CPT1A* to transcriptionally activate their expression and allow normal fatty acid oxidation function. Hepatic steatosis (right): Loss of GHR reduces the expression of AHR by reducing the phosphorylation level of ERK1/2. AHR downregulation directly reduces fatty acid oxidation, leading to hepatic steatosis.

In summary, *GHR* KO pigs showed abnormal lipid metabolism, especially high serum levels of FFAs and hepatic steatosis, consistent with the results observed in LS patients. A key mediator of this effect appears to be that *GHR* deficiency in hepatocytes inhibited the expression of AHR by decreasing the phosphorylation level of ERK1/2. Moreover, we confirmed that *ACOX1* and *CPT1A* were direct target genes of *AHR*. The reduction in AHR expression directlyled to a reduction in fatty acid oxidation, an important cause of hepatic steatosis in the context of *GHR* deficiency (Figure 9). Thus, AHR in hepatocytes is a critical factor in the initiation of hepatic steatosis and a potential molecular target for intervention.

## Supplementary Material

Supplementary figures and table.

## Abbreviations

LS: Laron syndrome
GHR: growth hormone receptor
FFAs: free fatty acids
ZFN: zinc finger nuclease
CDS: coding sequence
NC: negative control
HOMA-IR: homeostatic model assessment of insulin resistance
HOMA-IS: homeostatic model assessment of insulin sensitivity
TGs: triglycerides
TC: total cholesterol
HDL: high-density lipoprotein
LDL: low-density lipoprotein
ALT: alanine aminotransferase
AST: aspartate aminotransferase
DEGs: differentially expressed genes
GO: Gene Ontology
KEGG: Kyoto Encyclopedia of Genes and Genomes
TFs: transcription factors
ChIP: chromatin immunoprecipitation
Co-IP: Co-Immunoprecipitation
