# Supplementary material for: Systemic Deficiency of GHR in Pigs leads to Hepatic Steatosis via Negative Regulation of AHR Signaling
Source: Int J Biol Sci. 2021 Oct 3;17(15):4108–21. doi: 10.7150/ijbs.64894 (PMC8579453; doi:10.7150/ijbs.64894)
Supplement: Supplementary file 1 — Supplementary figures and table. [file ijbsv17p4108s1.pdf]

Systemic deficiency of GHR in pigs leads to hepatic steatosis via negative regulation of AHR signaling

Qi Han[1], Huiling Chen[1], Likai Wang[1], Yang An[2], Xiaoxiang Hu[1], Yaofeng Zhao[1], Hao Zhang[3], Ran Zhang[1] *

[1]State Key Laboratory for Agrobiotechnology, College of Biological Sciences, China Agricultural University, Beijing, 100193, China.

[2]MD Department of Plastic Surgery, Peking University Third Hospital, Beijing, 100191, China

[3]National Engineering Laboratory for Animal Breeding, China Agricultural University, Beijing, 100193, China.

*Corresponding author:

Ran Zhang, State Key Laboratory for Agrobiotechnology, College of Biological Sciences, China Agricultural University, Beijing, 100193, China.

Telephone: 86-10-62731327

E-mail address: zhangran0628@cau.edu.cn

Supplementary Figure 1

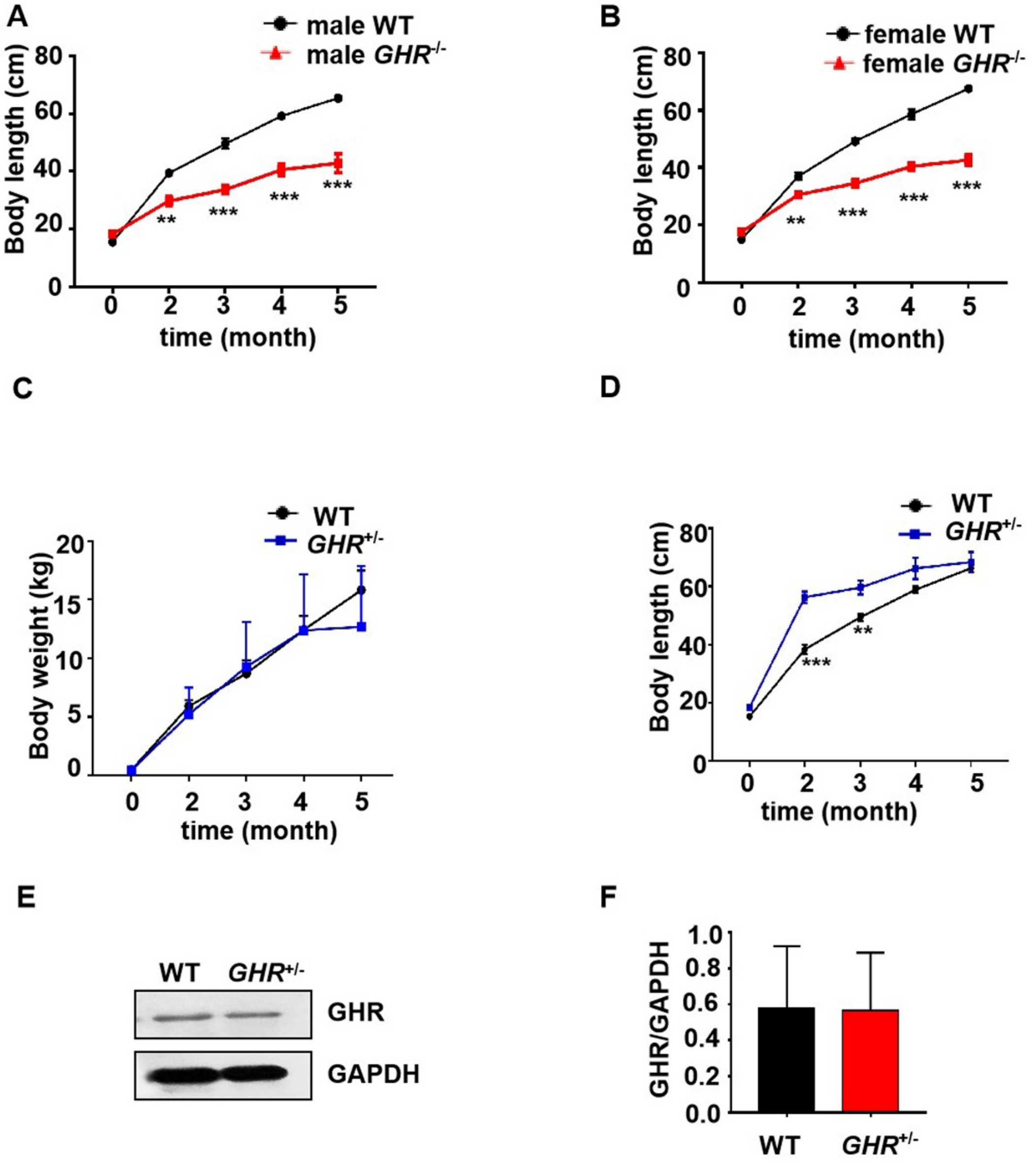

Supplementary Figure 1: Phenotype of GHR KO pigs. (A-B) Body length of male and female *GHR* KO pigs and their WT littermates. (C-D) Body weight and length of heterozygous GHR mutant and WT pigs. (E-F) Protein levels in GHR KO pigs. n =3 pigs per group. The data are presented as the mean ± SD values. *, P < 0.05; **, P < 0.01; ***, P < 0.001.

Supplementary Figure 2

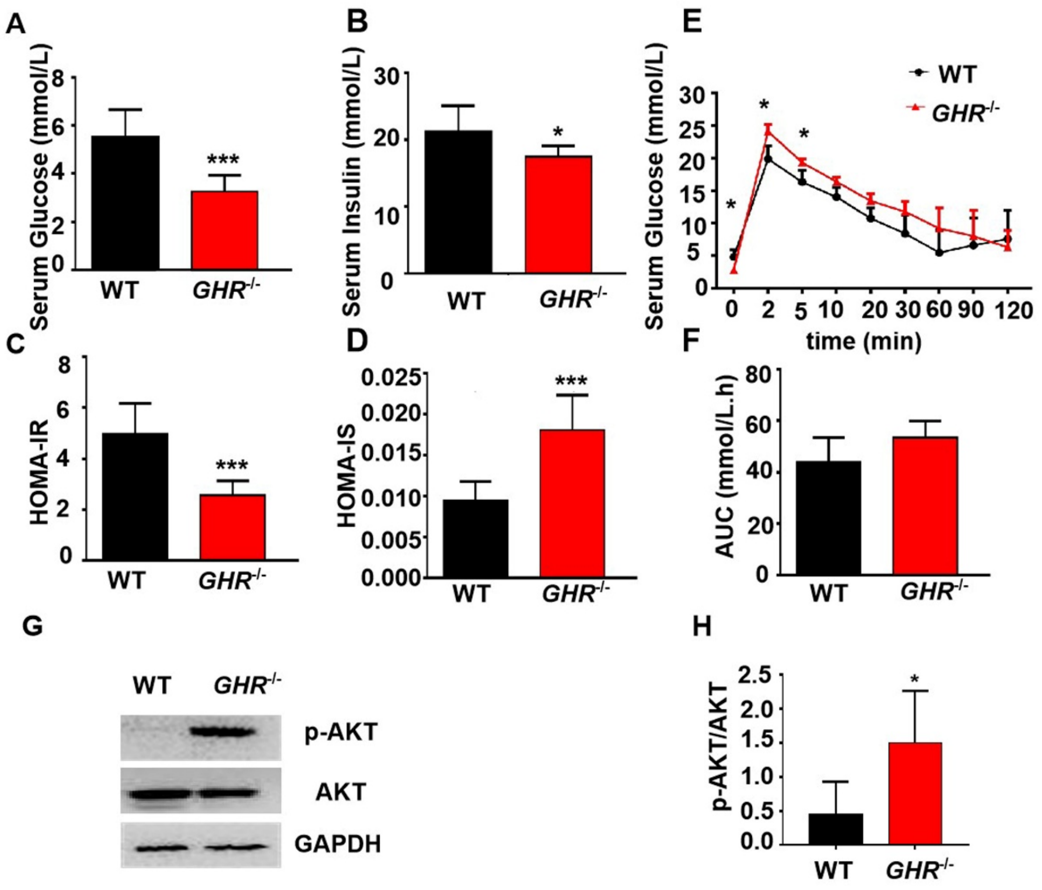

Supplementary Figure 2. GHR deficiency disrupted glucose/lipid homeostasis in pigs. (A) Fasting blood glucose levels in GHR KO pigs and their WT littermates. (B-D) Fasting insulin levels and HOMA-IS and HOMA-IR indexes for WT and GHR KO pigs. (E-F) GTT results for WT and GHR KO pigs and the corresponding areas under the curve (AUCs) for the GTTs. (G-H) Protein levels of p-AKT and AKT in pigs by Western blotting, and the content was quantified by ImageJ. n = 3 pigs per group. The data are presented as the mean ± SD values. *, $P < 0.05$; **, $P < 0.01$; ***, $P < 0.001$.

Supplementary Figure 3

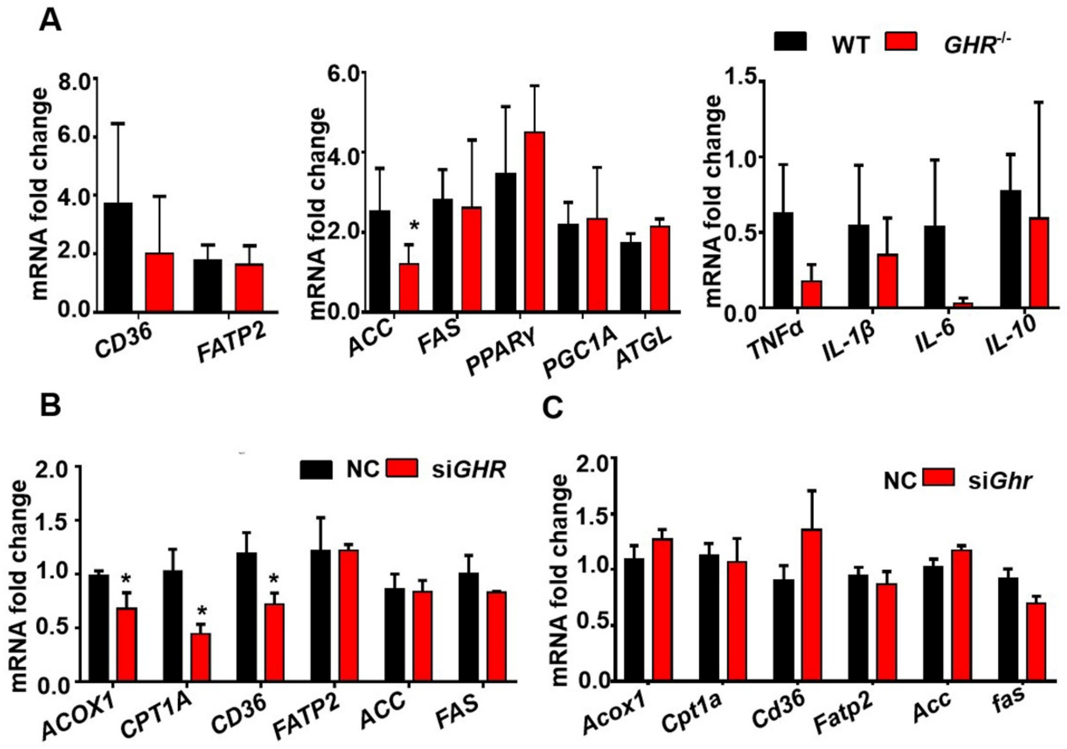

Supplementary Figure 3: (A) mRNA levels of fatty acid uptake and synthesis-related genes and inflammation-related genes in GHR KO pigs. (C) mRNA levels of fatty acid oxidation-, uptake- and synthesis-related genes in siGHR human hepatocytes. (D) mRNA levels of fatty acid oxidation-, uptake- and synthesis-related genes in siGhr mouse hepatocytes. Scale bar: 100 µm. n =3 pigs per group. The data are presented as the mean ± SD values. *, P < 0.05; **, P < 0.01; ***, P < 0.001.

Supplementary Figure 4

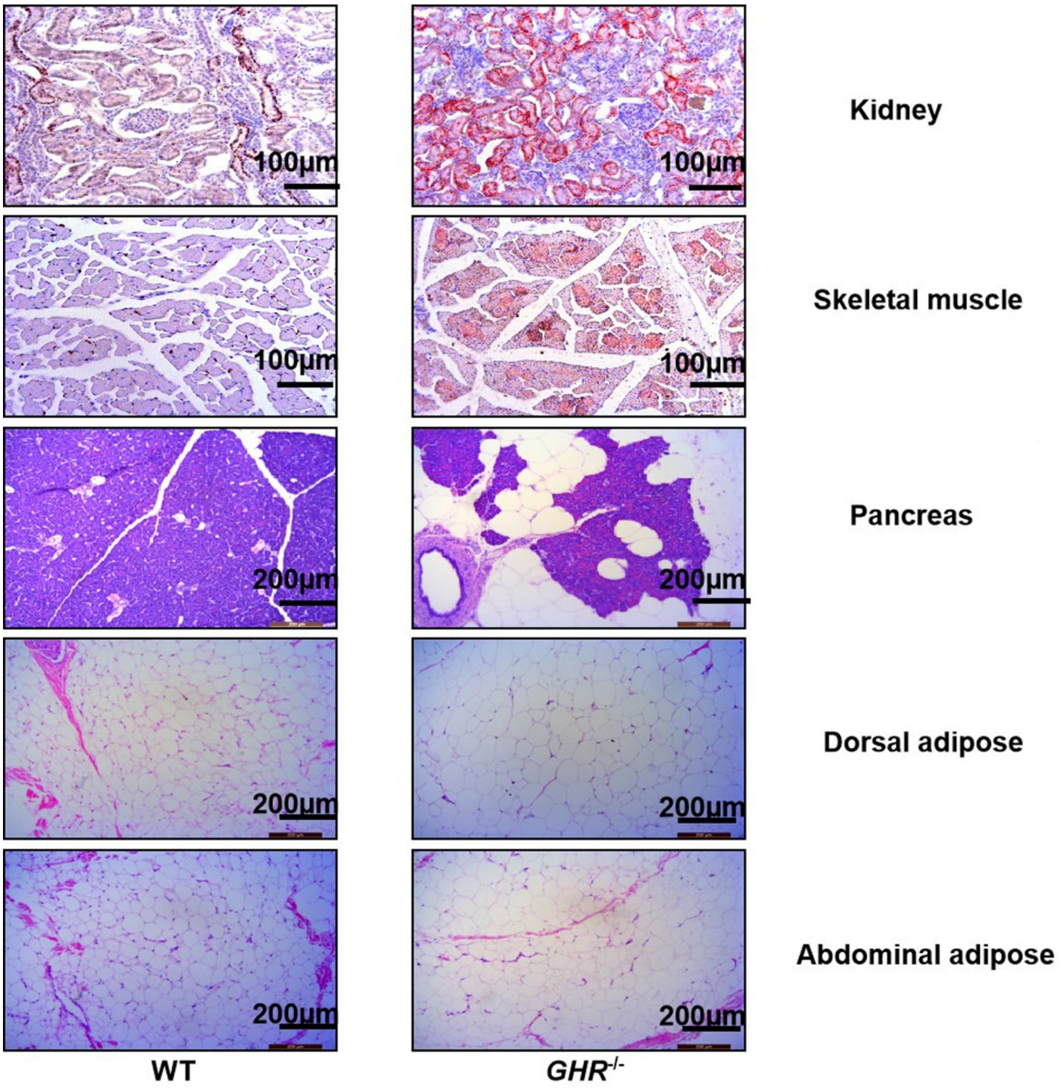

Supplementary Figure 4: Representative image of Oil red O staining. Scale bar: 100 μm. Representative image of H&E staining. Scale bar: 200 μm.

Supplementary Figure 5

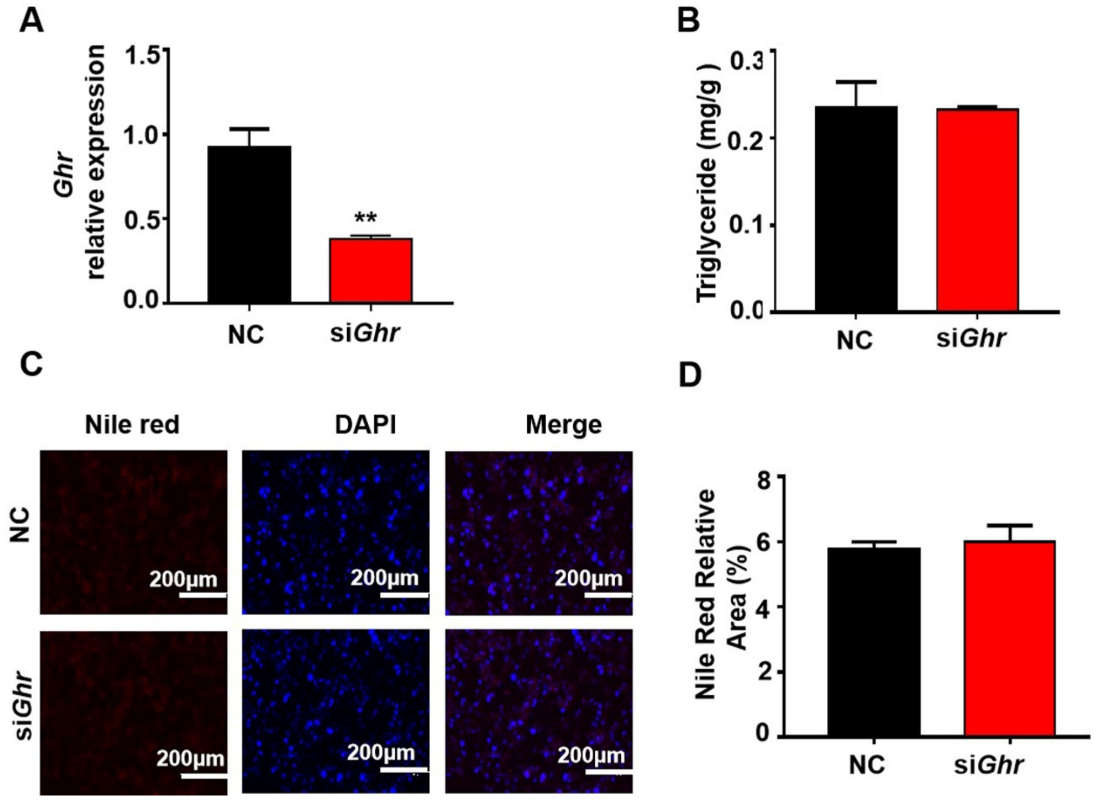

Supplementary Figure 5: *Ghr* depletion did not cause intracellular lipid accumulation in cultured mouse hepatocytes. (A-D) Hepatocytes were treated with NC or *Ghr* siRNA (si*Ghr*). (A) GHR mRNA expression levels in mouse hepatocytes. (B) TG levels in mouse hepatocytes. (D) Nile red staining of mouse hepatocytes with NC or si*Ghr*. (E)The neutral lipid content was quantified with ImageJ and normalized to the number of nuclei. Scale bar: 100 μm. n = 3 per group. The data are presented as the mean ± SD values. *, P < 0.05; **, P < 0.01; ***, P < 0.001.

Supplementary Figure 6

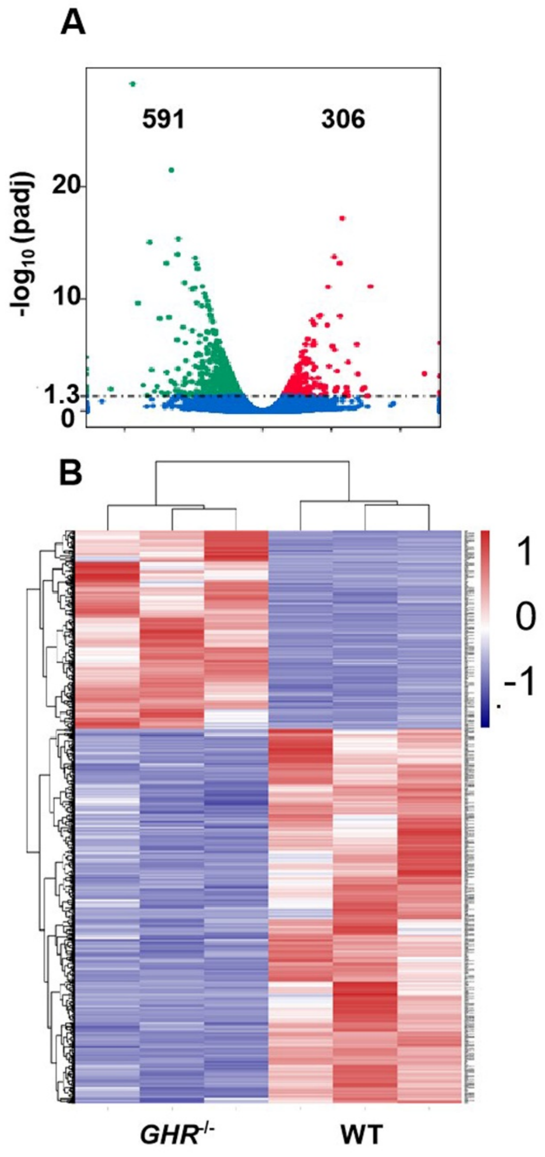

C

Associated_Gene_ Name	log_2FoldChange
TRIB3	3.2672
NFE2	3.1371
MAFF	3.1087
YBX3	2.5036
ATF3	2.2767
ATF4	1.8189
ETS2	1.6869
PP	0.6693
IRF6	-1.6112
PPARA	-1.6116
ZHX1	-1.7205
SOX4	-1.7885
PROX1	-1.7918
AEBP1	-1.8623
AHR	-2.1127
KAT2B	-2.1762
ZNF461	-2.2294
NR1I3	-2.5616
SOX9	-2.6033
ESR1	-2.964
AR	-3.4891
TP63	-5.456
RFX4	-5.9627

Supplementary Figure 6: Transcriptome analysis of hepatic gene expression profiles in *GHR* KO pigs. (A) Genome-wide changes in mRNA expression are shown in a volcano plot. (B) Hierarchical cluster analysis of the DEGs. Samples are displayed in columns and genes in rows. Gene expression levels are represented as colours, with brighter red for higher values and brighter green for lower values. (C) Transcription factors identified based on the DEGs.

Supplementary Figure 7

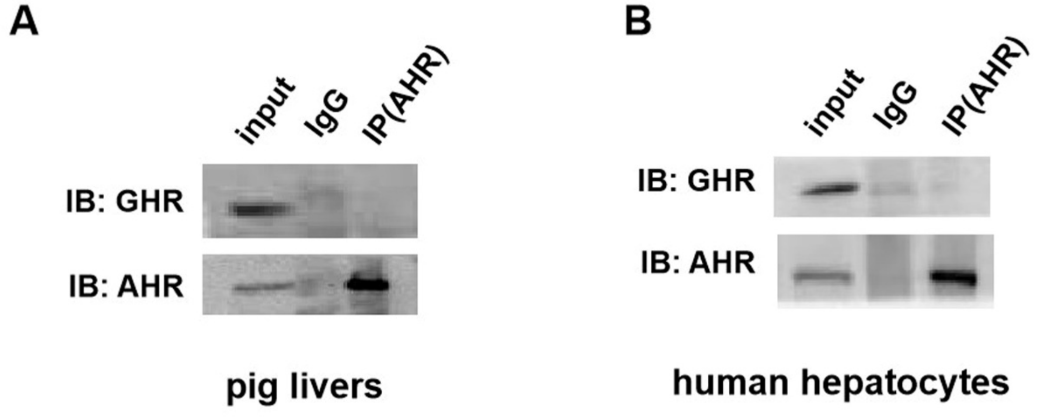

Supplementary Figure 7: AHR did not bind to GHR. (A) Pig livers were lysed and immunoprecipitation was carried out with AHR antibodies. The immunocomplexes were subjected to Western blot analysis. (B) Human hepatocytes were lysed and immunoprecipitation was carried out with AHR antibodies. The immunocomplexes were subjected to Western blot analysis.

Supplementary Table S1

The main primers of q-PCR

Gene	Sequences (Forward)	Sequences (Reverse)
GHR	GGATAAAGAGTATGAAGTGCGTGTG	GATAATTAAGAACCATGGAAACCGG
ACOX1	ACCACGGTGAAGAAGATAAGG	TGGCTCAGCAAGGTAGGAA
ACSL1	TCCTTGACAGTGATGAGCCCTTGG	TTCTGAGCGAAGATGCCGACGAA
CPT2	GGCTGCCTATTCCCAAACTTGAAGA	ATTCTGCTTGTCCTGAGCAACCAG
CPT1A	GGATGACGGCTCTGGCACAAGATT	GTCTGTAAAGCAGGATGGCGTGGAT
ACADM	AGGAGCCATTGATGTGTGC	CTGCTTTGGTCTTTATACCAGCTA
ACADS	GGAGGCTCAGGTGAAGAAG	GTGTAGGCCAGGTAATCCAG
ACADL	TGTCTCCAGCTGCATGAAACGA	AGCTGCACACAGTCATAAGCCA
ECH1	ACTGTCGTGCGGTGGTGATCTCT	CGATGACGCTGAAGGTCTCCTGGTA
HADH	TAGCCAATGCCACCACCAGACAG	CACCTCGTTCGTACAGCCTGACT
ACAA1	GCCTCCTTCCCTCCTGTTTCAGAA	TTCACTCGCCTCAGCATCCATCC
CYP7A1	TGGTGCCAATCCTCTTGAGTTCCT	GTCAATGCTTCTGTGCCCAAATGC
CYP27A1	GGCGATACCTGGATGGCTGGAATAC	GTCAGCGTGTTGGATGTCGTGTCT
EHHADH	TGGGTGCCTTGCTCAGGAGACTT	GCCGAGAATGCCAACAGAGGAGATG
CYP4A24	ATCCGCCAGTACCAGGAGTTAGCA	CCACAGCCACCTTCATCTCGTTCAT
hAHR	ATGTCGTCTAAGGTGTCTGCTGGAT	TGGATGGTGGCTGAAGTGGAGTAG
pAHR	GGACAGAGAATTGATGAAGCGAGTG	TGGATGGTGGCTGAAGTGGAGTA
mGHR	CTGATTTTACCCCCAGTCCC	ATGGTGTTCACCTCCTCCAAC
mAHR	GCAGAATCCCACATCCGCAT	AGGGCTGGAGATCTCGTACAAC
